# Evaluation of IL-6, FoxP3 Treg lymphocytes, intestinal barrier biomarkers and the use of synbiotics in obese adolescents: a pilot study

**DOI:** 10.3389/fped.2023.1215793

**Published:** 2023-10-04

**Authors:** Cylmara Aziz, Armando Morales, Walter Pinto, Vanessa Fanchini, Luis Dell Aquila, Carine Sangaleti, Rosilene Elias, Maria Dalboni

**Affiliations:** ^1^ Department of Postgraduate Studies in Medicine, Universidade Nove de Julho, São Paulo, Brazil; ^2^ Department of Postgraduate Studies in Nanosciences and Biosciences, Universidade Estadual do Centro Oeste, Guarapuava, Brazil

**Keywords:** obesity, adolescent, inflammation biomarker, intestinal barrier biomarker, synbiotic

## Abstract

**Aim:**

This prospective pilot study evaluated inflammatory and intestinal barrier biomarkers and the effects of a synbiotic in obese adolescents.

**Methods:**

Eighteen obese and 20 eutrophic adolescents were evaluated for body composition using bioimpedance analysis (BIA), body mass index (BMI), IL-6 and lipopolysaccharide (LPS) serum levels, CD4 and FoxP3 Treg lymphocytes and monocytes. Synbiotic supplementation for 60 days was also evaluated for these parameters only in obese adolescents.

**Results:**

We observed an increase in CD4 lymphocyte (18.0 ± 12.4 vs. 8.9 ± 7.5; *p* < 0.01), IL-6 (0.30 ± 0.06 vs. 0.20 ± 0.06; *p* = 0.02) and LPS (0.18 ± 0.15 vs. 0.08 ± 0.05; *p* < 0.01) levels in obese compared to eutrophic adolescents. After synbiotic supplementation, FoxP3 Treg lymphocytes increased (14.0 ± 6.7 vs. 9.9 ± 5.4; *p* = 0.02) in obese adolescents.

**Conclusions:**

Obese adolescents presented a state of microinflammation and intestinal barrier breakdown, and synbiotic supplementation increased the expression of FoxP3 Treg lymphocytes, an anti-inflammatory regulator. Whether the increase in FoxP3 Treg lymphocytes may have an impact on inflammation and outcomes in obese adolescents deserves further evaluation.

## Introduction

The largest global epidemiological study of underweight, overweight and obesity rates, involving more than 130 million people, showed that the number of children and adolescents affected by obesity has increased tenfold over the past four decades (1).

The impact of obesity on health and disease has been linked to the development of type 2 diabetes mellitus (T2DM) (2), hypertension, cardiovascular disease and cancer (3).

In the long term, obesity in childhood and adolescence is associated with increased morbidity and mortality in adulthood due to an increased risk of coronary heart disease and cancer (4–6). It has been reported that adolescents aged 12–19 years have the highest prevalence of obesity at 20.6%, compared to 18.4% for youth aged 6–11 years and 13.9% for children aged 2–5 years (7).

Similar to adult obesity, adolescent obesity promotes inflammation and increases the risk of developing chronic diseases into and throughout adulthood (8).

Recent evidence indicates that visceral obesity depends not only on the release of proinflammatory adipokines but also on the immune cell mechanisms present in adipose tissue (9). In lean individuals, visceral adipose tissue (VAT) is characterized by the presence of high levels of FoxP3 + regulatory T lymphocytes (FoxP3+ Tregs) and the expression of cytokines such as IL-4, IL-5, IL-10, and IL-13 (10). These factors contribute to the polarization of macrophages in the VAT from an M1 (proinflammatory) phenotype to an M2 phenotype characterized by anti-inflammatory activity. However, in obese individuals, the hypertrophied VAT is characterized by lower FoxP3+ Treg expression (11) and higher levels of CD8 and CD4 lymphocytes that secrete IFN-ɣ, favoring the polarization of macrophages to an M1 phenotype and producing cytokines with proinflammatory activity, including IL-1β, IL-6, and TNF-ɑ (12, 13).

Obese adolescents present elevated levels of IL-6 (14, 15), TNF-α and oxidative stress (16), but data on obesity and FoxP3+ Tregs in obese adolescents are scarce.

In recent years, the role of the gut in the development of obesity has been increasingly recognized. Obesity is associated with intestinal barrier dysfunction and dysbiosis, which contributes to the activation of local inflammation, contributing to systemic inflammation (17–19). Studies conducted with animal models provided evidence that inflammation may originate in the gut owing to modulation of gut barrier function leading to metabolic endotoxemia (20, 21) with an increase in gut-derived plasma lipopolysaccharide (LPS) levels, which may result in low-grade systemic inflammation (22). LPSs are an indicator of impaired intestinal epithelial barrier function, also known as “leaky gut” syndrome. Accordingly, studies have found high levels of gut-derived bacterial products in the blood of obese patients (23).

Some recent studies have shown that the use of prebiotics, probiotics, and synbiotics can contribute to restoring the intestinal microbiota, resulting in protection of the intestinal barrier and reduction of local and systemic inflammation (24, 25).

Thus, the objectives of this pilot study were to evaluate inflammatory, intestinal barrier, and immune cell biomarkers in obese adolescents and to assess the effects of synbiotic supplementation in these adolescents on the same parameters described above.

## Methods

### Study design

Phase 1 was a convenience sample cross-sectional study comparing obese and eutrophic adolescents with regard to BMI, metabolic parameters, immune cells, inflammation, and intestinal barrier biomarkers.

Phase 2 was a prospective longitudinal study that evaluated the effects before and after the use of synbiotic supplementation in the obese group on all the parameters described above.

A measurement at or above the 85th percentile and below the 95th percentile on the age- and sex-specific growth charts indicates overweight status, while a measurement at or above the 95th percentile indicates obesity (26).

Adolescents of both sexes aged 11–17 years were evaluated and classified according to the body mass index curve in the Z score, where the obese index = a Z score > + 2 SDs and the eutrophic index = a Z score ≤ + 1 SD. The interpretation of cutoffs was as follows: obese adolescents: > + 2 SDs (equivalent to a BMI > 25 kg/m^2^ from 11 to 17 years), and eutrophic adolescents: <1 SD >1 SD) (equivalent to a BMI > 16 kg/m^2^ and <20 kg/m^2^ from 11 to 17 years) (27).

Adolescents with a BMI less than 14 kg/m^2^ or greater than 40 kg/m^2^, genetic syndromes, hypothyroidism and/or type 1 diabetes, immunodeficiencies, degenerative diseases and eating behavior disorders (anorexia, bulimia, eating disorder not otherwise specified), use of dietary and/or medication treatment for obesity or dyslipidemia, or use of corticosteroids, anti-inflammatory or antibiotic drugs were not included in this study.

In addition, as usual during a medical consultation, a 24 h food recall, an anamnesis that included questions about drugs and medicines used, and a sleep inquiry were performed. Thirty-eight adolescents (11–17 years old) were included in this pilot study between June 2020 and March 2022: 18 were obese, with a BMI > 25 kg/m^2^ (obese group), and 20 were eutrophic, with a BMI > 16 kg/m^2^ and < 20 kg/m^2^ (eutrophic group).

All participants and their legal guardians signed informed consent forms. This study was approved by the Ethics Committee No. 2.962.852 CAAE: 93900718.0.0000.5511 [Universidade Nove de Julho (UNINOVE), Sao Paulo, Brazil], in accordance with the Declaration of Helsinki.

#### Phase 1

The adolescents underwent a psychiatric consultation for medical history, physical examination, measurements of weight, height, abdominal and cervical circumferences, blood pressure, and body composition by BMI and by bioelectrical impedance analysis (BIA). During the consultation, clinical aspects and pubertal staging of the adolescent were evaluated according to Tanner’s tables.

Five milliliters of peripheral venous blood was collected for the determination of total cholesterol, high-density lipoprotein (HDL), low-density lipoprotein (LDL), very low-density lipoprotein (VLDL), triglycerides, glycemia, glycated hemoglobin (HBA1c), insulin, proinflammatory cytokines (IL-6), and intestinal barrier biomarkers (LPSs). An additional 5-ml aliquot of peripheral venous blood was collected in a tube with anticoagulant (EDTA) for evaluation of FoxP3+ Tregs and monocytes.

#### Phase 2

Synbiotic supplementation: Only obese adolescents were provided with one (01) sachet/day for 60 days (post-intervention) of the synbiotic compound (Simbioflora®, Invictus Brasil FQM, SP, Brazil) (tasteless sachets containing 5.5 g of prebiotic (fruit oligosaccharide) and probiotics *Lactobacillus acidophilus* 109 CFU, *Lactobacillus rhamnosus* 109 CFU, *Lactobacillus paracasei* 109 CFU and *Bifidobacterium lactis* 109 CFU, without gluten, lactose or glucose). After 60 days of supplementation with Simbioflora, the following parameters were evaluated: BMI, BIA, FoxP3+ Tregs, monocytes, inflammation, and intestinal barrier biomarkers (LPSs).

### Anthropometric data

Body weight was measured and evaluated using an electronic scale (Seca®, Germany) with 100 g increments and expressed in kilograms (kg). The adolescent was instructed to stand upright, with arms at both sides, in the center of the weighted platform, wearing light clothing and no shoes and not holding any objects. Body mass index (BMI) was calculated as weight in kg divided by height in meters squared (kg/m^2^). Bioelectrical impedance analysis (BIA) is a method that uses a low-amplitude, high-frequency electric current passed through the body to estimate weight and total body fat percentage. BIA was performed with the adolescent lying on a hospital gurney, wearing light clothing and no shoes and not holding any objects. Body composition analyzers from RJL Systems (Michigan, USA) were used according to the manufacturer’s recommendations.

### Measurement of Il-6 and LPS serum levels

IL-6 was quantified by immunoenzymatic assay (ELISA) (minimum detection limit of 0.09 pg/ml) (HS600B, R&D Systems) according to the manufacturer’s recommendations. Gram-negative bacterial endotoxin and lipopolysaccharides (LPSs), were analyzed by the Limulus amebocyte lysate (LAL) chromogenic endpoint assay (minimum detection limit of 0.04 EU/ml) (HIT302, Hycult Biotech) according to the manufacturer’s recommendations.

### Measurement of Cd4 T lymphocytes (Cd4), Cd14 (M1), and Cd16 (M2) monocytes and FoxP3 regulatory T lymphocytes (FoxP3 Tregs)

Blood samples from the adolescents were collected in a tube containing EDTA; 100 μl was transferred to tubes for monoclonal antibody labeling. Briefly, 100 μl of blood was incubated for 30 min at +4°C with antibodies specific for cell surface antigens (PE-labeled anti-CD4, APC-labeled anti-CD14, and FITC-labeled anti-CD16 for CD4 lymphocytes and monocytes M1 and M2, respectively) (eBioscience). In the other tube, 100 μl of blood was treated with fixation/permeabilization buffer (eBioscience) at +4°C for 40 min and after incubation washed three times with permeabilization buffer to allow intracellular staining with APC specific for FoxP3 antibody (eBioscience) at +4°C for 30 min. After one wash, the cells were incubated with PE-labeled anti-CD25 and FITC-labeled anti-CD3 (eBioscience) for 30 min at +4°C. For both staining procedures, appropriate isotype-matched controls were used. Acquisition (100,000 events) and analysis of cell populations were performed by flow cytometry (ACCURI, Becton & Dickinson) and presented as the mean fluorescence intensity (MFI).

### Statistical analysis

The Kolmogorov‒Smirnov test was used to determine the normality of the data. Categorical variables are presented as absolute and relative frequencies. Student’s *t*-test was used to compare variables between two independent groups with a parametric distribution, and the Mann‒Whitney *U* test was used for nonparametric variables. The chi-squared test was used for the analysis of categorical variables. The paired *t*-test was used for the analysis of parametric samples at two time points, while the Wilcoxon test was used for nonparametric samples (before and after synbiotic supplementation). The significance level was set at 5% (*p* < 0.05), and the Spearman test was used to analyze correlations. Statistical analysis was performed with the Statistical Package for the Social Sciences (SPSS 25.0, IBM Sofware, USA) software.

## Results

As expected, we found that obese adolescents had a higher weight, neck and abdominal circumference, BMI, and percent fat mass (FM) and decreased fat-free mass (FFM), dry lean mass (DLM), and total body water as analyzed by bioimpedance (BIA) measurements compared to eutrophic adolescents. Although still within the normal range, the obese adolescent group showed an increase in systolic blood pressure compared to the eutrophic group (Table 1). In addition, all adolescents enrolled in this study reported having a sleep duration above 7 h a day and self-reported good quality of sleep. Furthermore, according to the 24 h food recall, all volunteers reported having a typical Brazilian diet, considering the timing of meals and the type of foods and amount of macronutrients (carbohydrates, proteins and fat).

**Table 1:** Anthropometric, bioimpedance and blood pressure results of eutrophic and obese adolescents pre-intervention.

Variables	Eutrophic group (*n* = 20)	Pre-intervention Obese group (*n* = 18)	*p*
Male	13 (65.0%)	11 (61.1%)	0.80^a^
Age (Years)	14 ± 1	14 ± 2	0.86
Weight (kg)	45.9 ± 6.9	83.4 ± 21.8	<0.001
NC (cm)	31.6 ± 2.1	36.5 ± 3.8	<0.001
AC (cm)	64.8 ± 4.3	95.9 ± 14.6	<0.001
BMI (kg/m^2^)	17.5 ± 1.9	31.0 ± 6.0	<0.001
FM (%-BIA)	13.8 ± 5.6	38.4 ± 5.1	<0.001
FFM (%-BIA)	86.2 ± 5.6	61.6 ± 5.1	<0.001
Total Body Water (%-BIA)	64.4 ± 5.0	45.6 ± 4.5	<0.001
Total DLM (%-BIA)	21.8 ± 1.9	16 ± 1	<0.001
BMI (kg/m^2^ BIA)	17.5 ± 1.9	30.6 ± 5.5	<0.001
SBP (mmHg)	101 ± 9	109 ± 9	0.009
DBP (mmHg)	69 ± 7	72 ± 8	0.16

Significance = *p* values < 0.05 in the unpaired Student’s *t-*test or chi-square test

^a^ NC, neck circumference; AC, abdominal circumference; BMI, body mass index; FM, fat mass; FFM, fat-free mass; DLM, dry lean mass; BIA, bioelectrical impedance analysis; SBP, systolic blood pressure; DBP, diastolic blood pressure.

Table 2 displays the drugs used by the adolescents enrolled in this study in both the eutrophic and obese groups.

**Table 2:** Description of the drugs used by the patients enrolled in this study.

Drugs used	Eutrophicgroup (*n* = 20)	Obese group (*n* = 20)
Metformin chlorhydrate (1,000 mg/day)	0	2
Gestodene (75 mcg) + Ethinylestradiol (30 mcg)/day	0	1
Dimesylate lisdexamfetamine (50 mg)/day	0	1
Sodium levothyroxine (50 mcg)/day	0	1
Nasal fluticasone (50 mcg)/day	0	1
Fexofenadine (120 mg)/day	1	0
Sodium montelukast (5 mg)/day	1	0
Oral immunotherapy (dust and mite)	2	0
Growth hormone (0,8 ml/day)	1	0
Sertraline (50 mg/day)	1	0
Isotretinoin (20 mg/day)	1	0

mg, milligram; mcg, microgram; mL, milliliter.

We observed high levels of insulin, triglycerides, VLDL cholesterol, CD4 lymphocytes, serum IL-6 and LPSs in obese adolescents compared to eutrophic adolescents (Table 3).

**Table 3:** Characteristics of metabolic variables, cellular immunity, inflammatory and intestinal permeability biomarkers of the eutrophic and preintervention obese groups.

Metabolic variables, cellular immunity and biomarkers	Eutrophic (*n* = 20)	Preintervention Obese (*n* = 18)	*p*
	Mean ± DP	Mean ± DP	
Glycemia (mg/dl)	87 ± 8	86 ± 8	0.68
HbA1c (mg/dl)	5.3 ± 0.3	5.5 ± 0.3	0.14
Insulin (mU/L)	9.6 ± 3.7	18.9 ± 8.9	0.02
Triglycerides (mg/dl)	71 ± 23	104 ± 45	0.01
Total Cholesterol (mg/dl)	149 ± 37	158 ± 30	0.40
LDL-c (mg/dl)	81 ± 30	90 ± 27	0.33
HDL-c (mg/dl)	51 ± 9	47 ± 6	0.18
VLDL-c (mg/dl)	15 ± 5	21 ± 9	0.02
TSH (ulU/ml)	2.27 ± 0.95	1.86 ± 0.93	0.22
Free T4 (ng/dl)	1.06 ± 0.18	1.07 ± 0.21	0.89
T-CD4^+^ (MFI)	8.9 ± 7.5	18.0 ± 12.4	0.01
FoxP3^+^ Treg *lymphocytes* (MFI)	9.9 ± 6.6	9.9 ± 5.4	0.99
M1-*CD14*^+^ *monocytes* (MFI)	69.5 ± 17.5	74.9 ± 15.3	0.32
M2-CD16^+^ monocytes (MFI)	24.4 ± 16.0	31.3 ± 22.7	0.28
IL-6 (pg/ml)	0.26 ± 0.06	0.30 ± 0.06	0.02
LPSs (EU/ml)	0.08 ± 0.05	0.18 ± 0.15	0.01

Significance =* p* values < 0.05 by Student’s *t*-test.

MFI, mean fluorescence intensity; EU, endotoxin units; IL-6, interleukin 6; LPSs, lipopolysaccharides.

Table 3 shows the biochemical data, cellular immunity, and inflammatory and intestinal barrier biomarkers in the eutrophic and obese groups pre-intervention.

No differences were observed in the anthropometric or body composition parameters assessed before and after the synbiotic intervention in obese adolescents (Table 4).

**Table 4:** Data on anthropometric, body composition and blood pressure values for obese adolescents pre- and post-supplementation with a synbiotic.

Variables	Pre-intervention	Post-supplementation	Post- vs. Pre-variation	*p*
BMI (kg/m^2^)	31.0 ± 6.0	30.3 ± 4.6	−0.7 ± 4.5	0.22
WC	95.9 ± 14.5	94.8 ± 14.3	−1.1 ± 3.5	0.18
NC (cm)	36.5 ± 3.8	36.5 ± 3.7	0.0 ± 0.7	0.92
Total body water (%-BIA)	45.6 ± 4.5	45.3 ± 4	−0.3 ± 1.8	0.54
FM (%-BIA)	38.4 ± 5.1	38.8 ± 4.7	0.3 ± 1.8	0.46
Total FFM (%-BIA)	61.6 ± 5.1	61.3 ± 4.7	−0.3 ± 1.8	0.46
Total DLM (%-BIA)	16.0 ± 1.0	16 ± 1.1	−0.1 ± 0.2	0.24
BMI (kg/m^2−^BIA)	30.6 ± 5.5	30.9 ± 5.5	0.3 ± 0.7	0.10
SBP (mmHg)	109 ± 9	108 ± 9	0 ± 8	0.88
DBP (mmHg)	72 ± 8	74 ± 7	2 ± 9	0.38

Significance = *p* values < 0.05 in the paired Student’s *t*-test or Wilcoxon test.

WC, waist circumference; NC, neck circumference; FM, fat mass; FFM, fat-free mass; DLM, dry lean mass; BIA, bioelectrical impedance analysis; BMI, body mass index; SBP, systolic blood pressure; DBP, diastolic blood pressure.

After supplementation with the synbiotic in obese adolescents, we observed a significant increase in CD4 lymphocyte (*p* = 0.002) and FoxP3 Treg (*p* = 0.02) levels, while the other parameters remained unchanged (Table 5).

**Table 5:** Data on inflammatory and intestinal barrier biomarkers in obese adolescents pre- and post-supplementation with a synbiotic.

Biomarkers	Pre-intervention	Post-intervention	Post- vs. Pre-variation	*p*
*CD4^+^ T lymphocytes* (MFI)	18.0 ± 12.4	29.6 ± 10.6	11.6 ± 13.6	0.002
*FoxP3 *^+ ^Treg *lymphocytes* (MFI)	9.9 ± 5.4	14.0 ± 6.7	4.1 ± 7.1	0.020
M1-*CD14*^+^ *monocytes* (MFI)	74.9 ± 15.3	72.6 ± 15.1	−2.3 ± 18.5	0.600
M2-CD16^+^ monocytes (MFI)	31.3 ± 22.7	41.6 ± 23.6	10.3 ± 30.2	0.160
IL-6 (pg/ml)	0.30 ± 0.06	0.32 ± 0.14	0.0 ± 0.1	0.550
LPSs (EU/ml)	0.18 ± 0.15	0.15 ± 0.12	−0.03 ± 0.12	0.280

Significance = *p* values < 0.05 in the paired *t*-test.

MFI, mean fluorescence intensity; EU, endotoxin units; IL-6, interleukin-6; LPSs, lipopolysaccharides.

In addition, we observed a significant positive correlation between BMI and SBP, DBP, CD4 lymphocytes, IL-6 and LPSs (Table 6). No differences were observed between the other variables.

**Table 6:** Analysis of correlations between BMI and blood pressure, CD4 lymphocytes, IL-6 and LPSs in obese adolescents.

Variable	Correlation coefficient (r)	*p*
SBP	0.64	<0.001
DBP	0.41	0.002
CD4 lymphocytes	0.39	0.003
IL-6	0.32	0.01
LPSs	0.56	<0.001

Statistical analyses were calculated using Spearman’s correlation coefficient.

Significance: *p* value ≤ 0.05.

BMI, Body mass index (kg/m^2^); SBP, systolic blood pressure; DBP, diastolic blood pressure; IL-6, interleukin-6; LPSs, lipopolysaccharides.

## Discussion

The present study showed that obese adolescents had a higher BMI and cervical and abdominal circumference compared to eutrophic adolescents. The obese adolescents showed a proinflammatory state and intestinal barrier dysfunction characterized by increased CD4 + lymphocyte, IL-6 and LPS levels. Synbiotic supplementation in obese adolescents increased FoxP3+ Treg lymphocytes, an anti-inflammatory modulator. As expected, BMI was higher in adolescents affected by obesity. To better assess lean and fat mass indices, BIA was used, and we found that fat mass was higher while fat-free lean mass and dry lean mass were lower in obese adolescents. Although we did not assess outcomes in this population in the present study, higher abdominal circumference and excess fat mass have been associated with inflammation, atherosclerosis, metabolic diseases, and diabetes mellitus (28, 29)*.* A higher concentration of insulin was observed in obese adolescents than in eutrophic adolescents. It has also been reported that fat mass correlates with increased insulin and other metabolic changes, such as triglycerides (30). However, except for a significant increase in VLDL-c in obese adolescents, we did not observe changes in the concentrations of total cholesterol or fractions of cholesterol.

As described above, obesity contributes to an inflammatory state. If an unhealthy dietary pattern is maintained, the increase in proinflammatory markers in obesity is a risk factor for the development of metabolic changes (3). In our study, we did not change the diet of the participants. We observed a higher serum concentration of IL-6 in obese adolescents, suggesting subclinical inflammation, probably due to hypertrophy of visceral fat.

The role of FoxP3+ Treg lymphocytes in obesity is still controversial and limited in human studies, especially in children. Similar to our findings, Calcaterra V et al. did not find a significant difference in Treg levels between obese and control groups (31). In contrast, Wen J et al. reported a significant decrease in FoxP3+ Treg lymphocytes in obese children (11). In studies among obese adults and children, FoxP3+ Treg lymphocytes were reduced (32, 33). Although we observed no difference regarding the predominance of M1 or M2 monocytes or FoxP3+ Treg lymphocytes between the groups studied, we found that obese adolescents had a higher expression of CD4 lymphocytes, suggesting that obesity may activate a greater proliferation of these cells and amplify the inflammatory state. Adolescents who had infections were excluded from this study, so the increase in CD4 and IL-6 levels seems to be associated with obesity. Reinforcing this hypothesis, a positive correlation between BMI and IL-6 and CD4 lymphocytes was also observed.

Recently, it has been reported that obese individuals have an imbalance in the microbiota (dysbiosis), which alters the intestinal barrier and contributes to local and systemic inflammation (34–36). Higher serum IL-6 levels are associated with microbiota imbalance, reinforcing the role of dysbiosis in microinflammation. In addition, dysbiosis contributes to changes and breakdown in the permeability of the intestinal epithelial barrier, resulting in an increased inflammatory response due to the translocation of pathogenic microorganisms or endotoxin (LPS) from gram-negative bacteria, an important factor in local and systemic inflammatory responses (37, 38).

We observed that obese adolescents showed a significant increase in serum LPS levels compared to eutrophic adolescents, which could be due to a breakdown of the intestinal barrier. However, this is a hypothesis because we did not investigate the microbiota of these adolescents.

The use of prebiotics, probiotics and synbiotics has been reported to restore the intestinal microbiota, which could contribute to less breakdown of the intestinal barrier and improve local and systemic inflammation (39, 40).

In the present study, we conducted an intervention with a synbiotic compound for 60 days in obese adolescents without dietary restriction to assess whether this supplement would modulate anthropometric, intestinal barrier and inflammatory biomarkers.

We observed no changes in BMI, BIA, IL-6 or LPSs after 60 days of synbiotic supplementation. However, we observed an increase in CD4 and FoxP3+ Treg lymphocytes. The high CD4 lymphocytes were probably due to obesity itself, and synbiotics were not able to downregulate them, at least during the 60 days of intervention.

In our study, we observed the upregulation of FoxP3+ Treg lymphocytes by synbiotic supplementation. FoxP3+ Treg lymphocytes contribute to anti-inflammatory systemic and local responses. In an experimental model of colitis, increases in FOXP3+ Treg lymphocytes were associated with preservation of intestinal mucosal integrity and an anti-inflammatory response (41). It is possible that modulation of FoxP3+ Treg lymphocytes in obesity may be a promising alternative treatment.

This study has some limitations. Our results should be interpreted with caution due to the small sample size and lack of inclusion of a group of obese adolescents who did not receive synbiotics. In addition, we did not control for confounding variables such as diet, timing of eating, or quality of sleep, which could have interfered with the results of the intervention with the synbiotic. On the other hand, we included 2 tables, demonstrating in Table 2 the drugs used by each of the participants and in Table 3 the caloric and macronutrient consumption of participants, to better characterize our patients. In addition, we described the sleep quality and duration of all participants. Despite these study limitations, it should be emphasized that only a few studies have evaluated LPSs as a biomarker of intestinal barrier breakdown and upregulation of FoxP3+ Treg lymphocytes by synbiotic supplementation in obese adolescents. To our knowledge, the current study is the first to evaluate these parameters in obese adolescents.

In conclusion, this study found that obese adolescents had a higher degree of inflammation (IL-6), with activation of CD4 + lymphocytes and a breakdown of the intestinal barrier (increase in LPS levels). The use of synbiotic supplementation increased the number of FoxP3+ Treg lymphocytes, so the use of synbiotics can be added as an adjunctive treatment until a normal BMI is achieved in obese adolescents with a proper nutritional diet. However, further studies should be conducted with a larger sample size and a control group composed of obese adolescents who do not take supplementation to investigate our hypothesis and the outcomes associated with obesity in adolescents.

## Data Availability

The raw data supporting the conclusions of this article will be made available by the authors, without undue reservation.
